# An Overgrowth Disorder Associated with Excessive Production of cGMP Due to a Gain-of-Function Mutation of the Natriuretic Peptide Receptor 2 Gene

**DOI:** 10.1371/journal.pone.0042180

**Published:** 2012-08-03

**Authors:** Kohji Miura, Noriyuki Namba, Makoto Fujiwara, Yasuhisa Ohata, Hidekazu Ishida, Taichi Kitaoka, Takuo Kubota, Haruhiko Hirai, Chikahisa Higuchi, Noriyuki Tsumaki, Hideki Yoshikawa, Norio Sakai, Toshimi Michigami, Keiichi Ozono

**Affiliations:** 1 Department of Pediatrics, Osaka University Graduate School of Medicine, Suita, Osaka, Japan; 2 Department of Orthopedics, Osaka University Graduate School of Medicine, Suita, Osaka, Japan; 3 Department of Cell Growth and Differentiation, Center for iPS Cell Research and Application, Kyoto University, Kyoto, Kyoto, Japan; 4 Department of Bone and Mineral Research, Osaka Medical Center and Research Institute for Maternal and Child Health, Izumi, Osaka, Japan; University of Iowa Carver College of Medicine, United States of America

## Abstract

We describe a three-generation family with tall stature, scoliosis and macrodactyly of the great toes and a heterozygous p.Val883Met mutation in *Npr2*, the gene that encodes the CNP receptor NPR2 (natriuretic peptide receptor 2). When expressed in HEK293A cells, the mutant *Npr2* cDNA generated intracellular cGMP (cyclic guanosine monophosphate) in the absence of CNP ligand. In the presence of CNP, cGMP production was greater in cells that had been transfected with the mutant *Npr2* cDNA compared to wild-type cDNA. Transgenic mice in which the mutant *Npr2* was expressed in chondrocytes driven by the promoter and intronic enhancer of the *Col11a2* gene exhibited an enhanced production of cGMP in cartilage, leading to a similar phenotype to that observed in the patients. In addition, blood cGMP concentrations were elevated in the patients. These results indicate that p.Val883Met is a constitutive active gain-of-function mutation and elevated levels of cGMP in growth plates lead to the elongation of long bones. Our findings reveal a critical role for NPR2 in skeletal growth in both humans and mice, and may provide a potential target for prevention and treatment of diseases caused by impaired production of cGMP.

## Introduction

Several lines of evidence indicate that signaling triggered by CNP plays an important role in chondrocyte development [1], [2]. Upon CNP binding, its cognate receptor natriuretic peptide receptor 2 (NPR2) functions as a guanylyl cyclase to increase cyclic guanosine monophosphate (cGMP) levels in chondrocytes, female reproductive organs, and endothelial cells [3], [4]. Transgenic mice that overproduce CNP exhibit excessive growth, while defects of the *CNP* or *Npr2* gene, leading to impairment of skeletal development [5]–[7]. The increase in cGMP level activates cGMP-dependent protein kinase II and seems to promote the accumulation of extracellular matrix in the growth plate of CNP-transgenic mice [8]. In human, overproduction of C-type natriuretic peptide (CNP) due to a chromosomal translocation was reported to cause skeletal dysplasia associated with tall stature [9]–[10]. In addition, acromesomelic dysplasia, type Maroteaux, characterized by dwarfism and short limbs, is caused by loss-of-function mutations in the *Npr2* gene [11]. On the other hand, NPR3, which is thought to act as a clearance receptor, knock-out mice resemble CNP transgenic mice [12].

In this paper, we describe the first family with tall stature and macrodactyly of both great toes caused by a gain-of-function type mutation in the *Npr2* gene. The mutant receptor, p.Val883Met, constitutively generates cGMP *in vitro*. Animal studies using the transgenic mice expressing the mutant NPR2 in chondrocytes demonstrated that skeletal overgrowth was associated with the overproduction of cGMP in cartilage. Our findings provide evidence that cGMP production downstream CNP/NPR2 system regulates the proliferation and differentiation of chondrocytes and determines skeletal growth.

**Figure 1:**
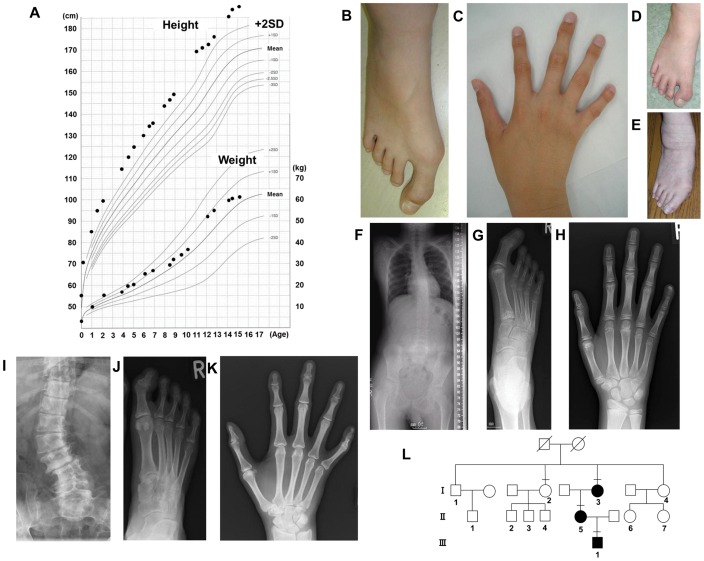
Clinical manifestations of the family. (**A**) Growth curve of the proband. The X- and Y- axis indicate age (year) and height (cm) or weight (kg), respectively. The bold line represents the mean for Japanese males. (**B, C**) Photographs of the proband’s right foot (B) and hand (C). The great toe was markedly long and wide. The fifth distal phalanx of the hand also showed minor clinodactyly. He has given written informed consent to publication of his photographs. (**D, E**) The right foot of the mother (D) and grandmother (E), respectively. The mother’s great toes were surgically shortened at 15 years of age. The grandmother’s great toes were large in early life and shortened due to arthrogryposis with aging. They have given written informed consent to publication of their photographs. (**F–H**) Radiographs of the proband’s skeleton. His spine showed mild scoliosis (F). The great toe was markedly long and wide (G), and the fifth distal phalanx of the hand showed minor clinodactyly (H). (**I–K**) Radiographs of the mother. Severe scoliosis and lumbar vertebra fractures (I) as well as a markedly long and wide great toe (J), and minor clinodactyly in the fifth digit of the hand (K) were observed, like in the proband. (**L**) Family tree. III-1, II-5, and I-3 indicate the proband, mother, and maternal grandmother, respectively, who had the phenotype. I-2 indicates a maternal grand-aunt, who had no phenotype and was included in the present study.

## Results

### Case Description

The proband was a 15-year-old boy born to non-consanguineous parents. He was born at 40 weeks gestation by spontaneous labor after an uneventful pregnancy. Birth parameters were as follows: weight, +0.78 SD; length, +3.1 SD; and head circumference; −0.2 SD. Long and wide great toes were already present at birth. His height remained above +2 SD (Fig. 1A). His psychomotor development was normal for his age. Fractures at the age of 11 and 12 years led to his referral to our unit. At that time, his height was 177.0 cm (+2.7 SD). His weight and arm span were in the normal range, +0.8 SD and 99.7% of height, respectively, and his stature was proportional. Blood pressure was normal. Physical examination showed tall stature, scoliosis, and long hands and feet with arachnodactyly of all the fingers and toes, the great toes being markedly long and wide. In addition, the fifth distal phalanx of the hands exhibited minor clinodactyly (Fig. 1B, C). He had no history of cardiac diseases, hypotension, ophthalmic disease, deafness, or digestive system disease. Hematological, biochemical and endocrinological values including insulin-like growth factor-I (IGF-I) were normal. However, bone formation and resorption markers were increased (serum bone-specific alkaline phosphatase, 270 U/l [normal range, 13–33.9]; osteocalcin, 12.5 ng/ml [normal range, 2.9–12.3]; cross-linked C-terminal telopeptide of type I collagen, 14.3 ng/ml [normal range, <4.5]; urinary cross-linked N-telopeptide of type I collagen, 524 nmolBCE/l [normal range, <55]). The bone mineral density (BMD) Z-score of the lumbar spine for L_2–4_, determined with dual-energy x-ray absorptiometry (Discovery A, Hologic), was −1.2, and −3.9 when corrected for his height. Bone age was 11.0 years. Radiological examination of the skeleton showed mild scoliosis, markedly long and wide great toes, and minor clinodactyly in the fifth digit of the hands (Fig. 1F–H). At the age of 15, his height reached 191.2 cm (+3.89 SD), and penile Tanner’s stage was IV.

**Figure 2:**
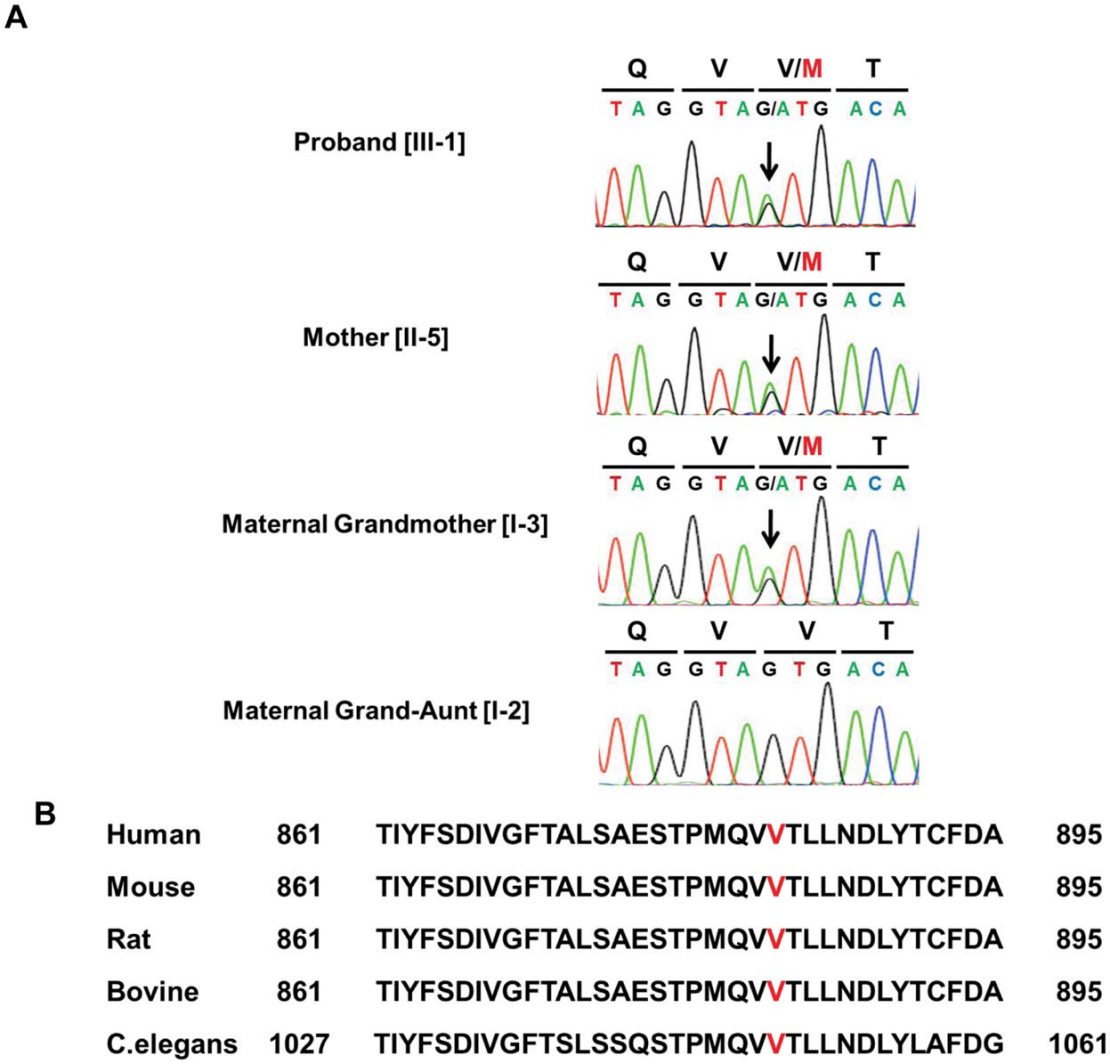
Identification of the *Npr2* mutation. (**A**) A novel G→A missense mutation at nucleotide +2647 creates a substitution, methionine for valine, at codon 883 in a heterozygous state in the proband [III-1], mother [II-5], and maternal grandmother [I-3] (arrows). The same position in the maternal grand-aunt without the phenotype [I-2] was not mutated. (**B**)Amino acid alignment of NPR2 among various species. Valine at codon 883 is located in a highly conserved region in the carboxyl-terminal guanylyl cyclase domain of NPR2.

The family tree (Fig. 1L) illustrates that the proband [III-1] has no siblings, and that his mother [II-5] and maternal grandmother [I-3] have the same phenotype (Fig. 1D, E). Clinical manifestations of II-5 were as follows: age, 46 years; height, 176 cm (+3.8 SD for her age); blood pressure, 140/70 mmHg. Her great toes were surgically shortened at 15 years of age. She was given no medication, including antihypertensive drugs. Her menstrual cycles are regular. Radiological examination of II-5 showed severe scoliosis and lumbar vertebra fractures as well as markedly long and wide great toes, and minor clinodactyly in the fifth digit of the hands, like the proband (Fig. 1I–K). The BMD of the lumbar spine for L_2–4_ could not be evaluated due to compressed fractures of the lumbar vertebrae. Clinical manifestations of I-3 were as follows: age, 63 years; height, 166 cm (+3.0 SD for her age); blood pressure, 134/70 mmHg. She underwent right hip replacement arthroplasty at 50 years of age because of degenerative hip disease. She developed hypertension up to 210/110 mmHg at 59 years of age, when antihypertensive therapy was initiated. Her great toes were large in early life and shortened due to arthrogryposis with aging.

**Figure 3:**
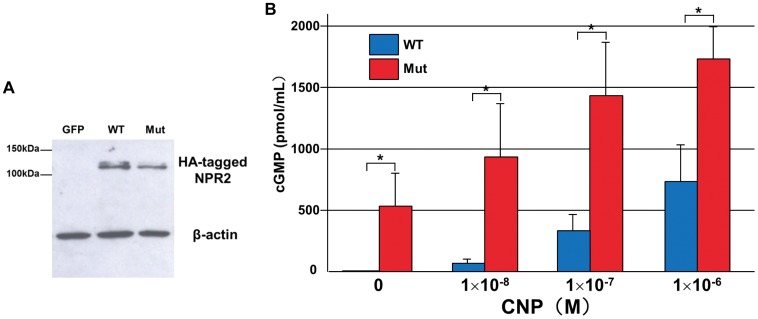
p.Val883Met is a gain-of-function mutation. (**A**) Western blot analysis confirmed the comparable expression of HA-WT (WT) and HA-Val883Met (Mut). HEK293A cells were transfected with HA-WT or HA-Val883Met constructs, and lysates were harvested for Western blotting using anti-HA antibody. As an internal control, β-actin in each sample was detected with anti-β-actin antibody. (**B**) Increased cGMP production in the HEK293A cells transfected with the p.Val883Met mutant (Mut) compared to that in wild-type cells (WT). Forty-eight hours after the transfection, the cells were serum-starved for 24 h, and then treated with the indicated concentrations of CNP-22 for 10 min, before cGMP production was assayed. Results are presented as the mean ± SD (N = 3, *p<0.05).

### Identification of a Novel Missense Mutation p.Val883Met in the Npr2 Gene

Since the phenotype of the patients most closely resembled several cases of the CNP overproduction phenotype due to a chromosomal translocation, in terms of tall stature and large great toes [9]–[10], enhanced CNP/NPR2 signaling was suspected. The concentration in serum of amino-terminal pro CNP was slightly decreased in III-1; 3.02±1.15 pmol/l, compared to the control (7.54±1.50 pmol/l). Those in II-5 and I-3 were almost in the normal range; 2.29 and 4.99 pmol/l, respectively, compared to the control subjects (3.53±0.75 pmol/l), ruling out overproduction of CNP. Since the proband’s phenotype also showed similarity to CATSHL syndrome [13], caused by a loss-of-function mutation in the *Fgfr3* gene, except for the absence of neurological symptoms, the *Fgfr3* gene was analyzed as well as the natriuretic peptide precursor C (*Nppc*), *Npr2*, and *Npr3* genes.

**Figure 4:**
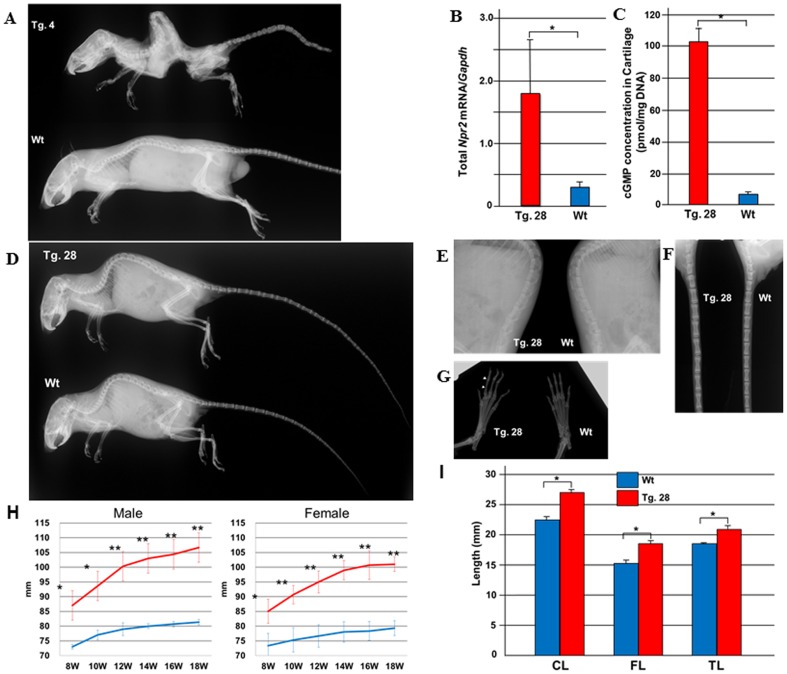
Transgenic expression of the p.Val883Met mutant in chondrocytes reproduced the patients’ phenotype in mice. (**A**) Radiographs of transgenic founder No. 4 expressing the p.Val883Met mutant NPR2 with the severest phenotype (Tg. 4) and a wild-type mouse (Wt). Tg. 4 exhibited severe bone deformities including elongation of the spine with severe kyphosis, metatarsal bones and distal phalanges, and died at seven weeks of age. (**B**) The total amount of *Npr2* mRNA in costal cartilage of line No. 28 offspring (Tg. 28) and wild-type littermates (Wt) at 5 days of age. The expression of *Npr2* mRNA in Tg. 28 was 5.1 times that in Wt. Results are presented as the mean ± SD (N = 3, *p<0.05). (**C**) Concentration of cGMP in costal cartilage of Tg. 28 and Wt at 5 days of age. The cGMP concentration in Tg. 28 was 17.3 times that in Wt. Results are presented as the mean ± SD (N = 3, *p<0.05). (**D–G**) Radiographs of Tg. 28 with a moderate phenotype and Wt at the age of 8 weeks. Body length, defined as the distance between the incisor and the anus, was longer in Tg. 28 than Wt. Total tail length was also longer in Tg. 28. The length of spinal (E) and tail (F) vertebrae was longer in Tg. 28 as well. All the metatarsal bones and distal phalanges were longer in Tg. 28 than Wt (G, arrows), and minor clinodactyly was detected, (**H**) The tail length of Tg. 28 (red line) and Wt (blue line) from 8 to 18 weeks. Tail length was always significantly longer in Tg. 28 than Wt among both males and females. Results are presented as the mean ± SD (N = 3, *p<0.05, ** *p*<0.001). (**I**) Bone length of Tg. 28 and Wt (18 week-old males). CL, Naso-occipital length of the calvarium; FL, femoral length; TL, tibial length. Results are presented as the mean ± SD (N = 3, *p<0.05).

We screened these four genes in III-1, II-5, I-3, and a maternal grand-aunt [I-2] who had no phenotype using genomic DNA extracted from peripheral blood leukocytes, and identified a novel heterozygous G→A missense mutation at nucleotide +2647 (c.2647G→A) of the *Npr2* gene in III-1, II-5, and I-3 that creates a substitution of methionine for valine (p.Val883Met), while the same position in I-2 was not mutated (Fig. 2A). This variant was not registered in the dbSNP (http://www.ncbi.nlm.nih.gov/projects/SNP/) and JSNP (http://snp.ims.u-tokyo.ac.jp/) databases nor found in 214 alleles from healthy Japanese controls. Amino acid Val883 is located in a highly conserved region of the carboxyl-terminal guanylyl cyclase domain of NPR2 and is conserved across species (Fig. 2B). No mutations were found in the *Fgfr3*, *Nppc*, and *Npr3* genes.

### Increased Plasma cGMP Concentrations in the Patients

The plasma concentrations of cGMP in 10 healthy adults were 3.1±1.1 pmol/ml. Plasma cGMP concentrations were elevated in III-1, II-5, and I-3; 20.0, 12.0, and 7.3 pmol/ml. Conversely, that in I-2 without the phenotype was 3.6 pmol/ml, which was similar to the values in healthy adults.

**Figure 5:**
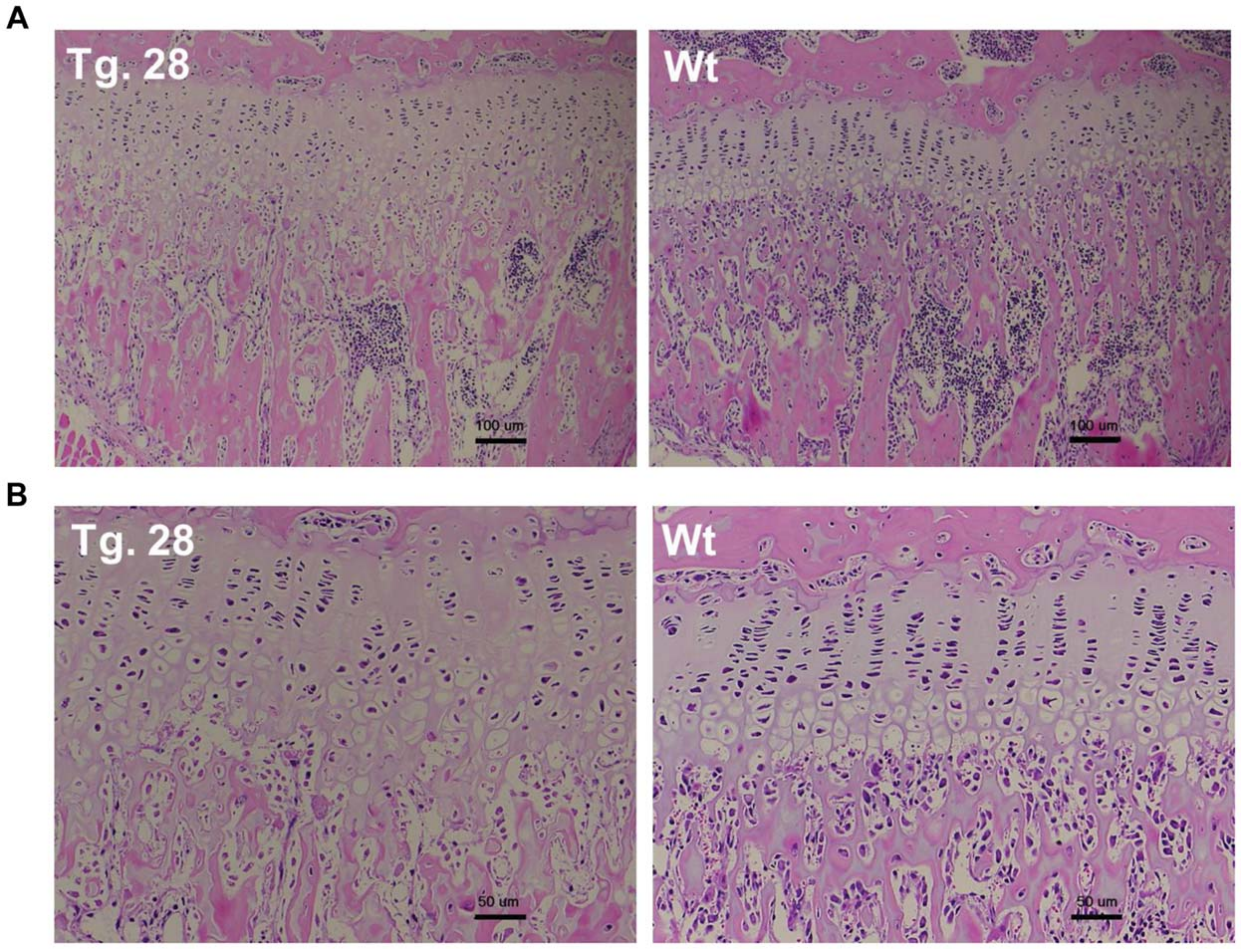
Hematoxylin-eosin-stained sections of growth plates of Tg. 28 and Wt at 8 weeks of age. The magnifications are ×40 (scale bar = 100 nm) and ×200 (scale bar = 50 nm) in (A) and in (B), respectively.

### p.Val883Met is a Gain-of-Function Mutation

To investigate the pathogenic significance of the p.Val883Met mutation, an *in vitro* functional assay was performed. HEK293A cells were transfected with the HA-WT and HA-Val883Met constructs, and whole-cell extracts were harvested for Western blotting. The Western blot analysis using anti-HA antibody confirmed that HA-WT and HA-Val883Met were expressed at comparable levels, with an approximate molecular size of 120 kDa (Fig. 3A). cGMP production in the cells expressing HA-WT and HA-Val883Met was also examined. Treatment with CNP-22 at a dose of 1×10^−8^–1×10^−6^ M increased intracellular cGMP levels in a dose-dependent manner with concentrations always significantly higher in HA-Val883Met-expressing cells than HA-WT-expressing cells (*p<0.05) (Fig. 3B). Intriguingly, cGMP was produced in HA-Val883Met-expressing cells even in the absence of CNP, while no production was observed in HA-WT-expressing cells. These results indicate that p.Val883Met is a gain-of-function type mutation.

**Table 1:** Primer sets for the human *Nppc* gene.

	Primer sequence (5′→3′)		
Exon	Forward	Reverse	Product size (bp)
1	GATTATAAAGGCGCGAGCAG	CTGCCTTCCCTCTCTCCTG	366
2	CTTGGGAGGGACACCCC	CACAACTTGAGCAAAGGCG	517

**Table 2:** Primer sets for the human *Npr2* gene.

	Primer sequence (5′→3′)		
Exon	Forward	Reverse	Product size (bp)
1–1	CTGTAGGCCAGAGCAGCC	AACCAGAGGCCACAGCAC	441
1–2	GACCCCGACCTGCTGTTAG	AGCTTTAGGGACACAGCGAC	454
2	GGGACATCCCAGTGATTCAG	ACAATTCCTGCCTGGTTCC	397
3	AAGTTTCCCTGTCCTGCATC	AGAGAAGCCCTTCCCCAAAG	253
4, 5	ACCCCAGAGAGAGGGGAAG	ACTGAACCCTTGAAAGCTGG	593
6, 7	GAAACAGCTGGGGTCTGG	CTTCAGCCAGAGGTTGTGTG	581
8, 9	AACCCTGCTGTTGGCTTG	GATGGGGATGAGACAGGATG	484
10, 11	CCACTGCTCTCTAGCATTTCC	CAGTATAACTTTGGGTCTATCCTTCC	540
12	ATGCTGGGTGATAGCTGGTG	CCACAATCAAAGAACAGTGGAAG	196
13, 14	AGCTAGCCAGTGCCCATCTC	TCTCTGAGGGTGATCAAAGG	640
15, 16	TACCCACAGCCTCTTCTTCC	GATGAGGCAGAGCCCAAC	602
17, 18	TGCAGCCACATACACTTTCC	TGAGTTTCAAAGCGGGTCTC	516
19, 20	GTGTCAAGCTTGTCTCCCTC	GGGATTATGAAAAGAAGAAAAGGG	496
21, 22	TCGGGCACGGTGCTATAC	GTGTGTGCAGACATGGTGG	458

**Table 3:** Primer sets for the human *Npr3* gene.

	Primer sequence (5′→3′)		
Exon	Forward	Reverse	Product size (bp)
1–1	AAGAGTGGGGAGGAAAGAGG	ACTGGCGCTGCTGCATAC	512
1–2	CTTCAGCTTGGTGGACCG	CTGCAGAGTGGACGAGTGTG	513
2	TGCTAGGTTCCTCCATGGTC	CACCACCCCTAGAAGTCCC	599
3	GAATGGCCTCTTTATGGTGG	AATGGCTCAGAATGGGACAC	318
4	CCTGGCATGAGTCACTTGG	CAGCTGCAACTGAGAAATACAG	272
5	TTTTGTTGGCAGACAGAGG	CAAATGCATTCCTCAGAGTCC	225
6	GGAATAGCTGTGGAAGGCTG	CTTACATTACACTGAGGTTAGAGGTG	276
7	CTCCAATGGGAAATCGAGG	CTGGGTGAAAGAGGGATGTG	293
8	AAGAGAAACCATGGTATTTTGTAGC	CATTGATGTCTTTCAACCCG	246

**Table 4:** Primer sets for the human *Fgfr3* gene.

Exon	Primer sequence (5′→3′)		
	Forward	Reverse	Product size (bp)
1	CGAGGGGGCGTGCCCTGCGCC	AGCACCGTTGGACCCCTCCG	339
2	AGGGGTCGGGACGCAGGAG	CCCAACGCCTCTGCCCGCAC	350
3	GTCTGTAAACGGTGCCGG	ACCAGAGAGACCCCCAGC	425
4	ATCTGGGAGGGGCACCTGGG	GTCCCTCAGCTGCCTGTGAAG	222
5	GTTCAGAGGGGCCTCTGCTC	AGTGAGCGGAGGCAGCAACC	290
6, 7	CTGCCTCCGCTCACTCAC	GAAGCTCCAACCCCTAGACC	554
8	TCTCCCACATCCTGCCTC	GGGCCTTGGAGCTGGAGCTC	277
9	AGGGCGGTGCTGGCGCTCGC	AGACAGTGCGGAGCAGCAGC	228
10	CAGGCCAGGCCTCAACGCCC	AGGCCTGGCGGGCAGGCAGC	271
11	CTGTACCTCCACGCCCTGTCGC	CTGTTTCACCCCCACCACC	264
12	GAGTGGGCGAGTTTGCACACTC	GTGCAGAGCAGGGCTGGGGGC	211
13	GTGCAGAGCAGGGCTGGGGGC	GCTCCTCAGACGGGCTGCCAG	240
14	CTGGCAGCCCGTCTGAGGAGC	CTGCTCCCAGCATCTCAGGGCA	286
15	GGTGGAGAGGCTTCAGCCCT	GCCAGGCGTCCTACTGGCATGA	217
16	TCATGCCAGTAGGACGCCTGGC	GGTCCTGGCTCTGCCCAGTTC	184
17	CAGCGCAGCCCTGGCCTATTC	CCTGAAGGGCTGCCAGTCCCT	314
18	GAAGCGGCGGGGCTCACTCCT	ATAGGCGGGTGGCACCAGGC	180
19	GCGAAGAGGGGCTCGGTGGCAC	CACCAGCAGCAGGGTGGGCTGCTAG	254

### Transgenic Expression of the p.Val883Met Mutant Npr2 in Mice Resulted in a Skeletal Phenotype Similar to that Observed in the Patients

Then, we further investigated the effects of p.Val883Met on skeletogenesis by generating transgenic mice expressing the mutant *Npr2* in chondrocytes. Transgenic founder No. 4 exhibited severe bone deformities including elongation of the spine with severe kyphosis, metatarsal bones, and distal phalanges, and died at seven weeks (Fig. 4A). Founder No. 28 also exhibited an elongated tail and toes with mild kyphosis, and could produce offspring mice. The amount of transgene mRNA in cartilage was quantified by real-time PCR and standardized based on the mRNA level of *Sox9*, a marker for chondrocytes. The higher expression of the transgene mRNA was detected in No. 4 than in No. 28 (data not shown).

**Table 5:** Primer sets used for the analyses of transgenic mice.

	Primer sequence (5′→3′)		
Name	Forward	Reverse	Product size (bp)
SV40SD/SA-HA	CTAGGCCTGTACGGAAGTGTTAC	GTAATCTGGAACATCGTATGGGTA	154
Npr2–SV40 poly(A)	TGTGGAAATGAAGGGAAAAGG	TCACTGCATTCTAGTTGTGGTTTG	149
Sox9	ACCCGCATCTGCACAACGCGGA	GGCTGGTACTTGTAATCGGG	182
Npr2	TTTCCGGCCAAGCATT	GAGGTTGTCCAATATGCTGGT	164
Gapdh	CCCGTAGACAAAATGGTGAAG	ATGGCAACAATCTCCACTTTG	104

SV40SD/SA-HA and Npr2-SV40 poly(A) were used to amplify the fragment derived from the transgene.

Then, we evaluated the skeletal phenotype in No. 28 offspring (Tg. 28). First, the expression of *Npr2* was determined using RNA extracted from costal cartilage of Tg. 28 and wild-type littermates (Wt) at 5 days of age. The total amount of *Npr2* mRNA, including native and transgenic *Npr2*, was quantified by real-time PCR and standardized based on the mRNA level of *Gapdh* (Tg. 28, 1.83±0.77; Wt, 0.36±0.08, *p<0.05) (Fig. 4B). cGMP concentrations in costal cartilage were also measured (Tg. 28, 102.2±11.3; Wt, 5.9±0.6 pmol/mg DNA, *p<0.05) (Fig. 4C). Although the total amount of *Npr2* was 5.1 times higher in Tg. 28 than Wt, the cGMP concentration was 17.3 times higher, supporting the *in vitro* data demonstrating the gain of function of the p.Val883Met mutant NPR2.

Representative radiographs of Tg. 28 (female, 8-week-old) are shown in Fig. 4D–G. The spinal and tail vertebrae and phalanges were longer in the transgenic mouse than Wt, indicating the longitudinal overgrowth of bones. Body length (data not shown) and tail length (Fig. 4H) were significantly longer in Tg. 28 than Wt from 8 to 18 weeks. In adulthood, Tg. 28 came to exhibit severe kyphosis and claudication in the legs, resembling the symptoms observed in patient I-3 and transgenic founder No. 4. In addition, naso-occipital length of the calvarium, femoral length, and tibial length were significantly longer in Tg. 28 than Wt at 18 weeks (*p<0.05) (Fig. 4I). Thus, the transgenic mice exhibited the skeletal overgrowth associated with the overproduction of cGMP in cartilage.

### Transgenic Mice Expressing the p.Val883Met Mutant Npr2 Exhibited Wider Growth Plates than Wild-type Littermates

Histological examination of the femora and tibiae in 8-week-old mice revealed that Tg. 28 had wider growth plates than Wt (Fig. 5A). At higher magnification, a disorganized arrangement of chondrocytes with increased number of hypertrophic chondrocytes was observed in Tg. 28 (Fig. 5B).

## Discussion

Here we have described a three-generation family with tall stature and macrodactyly due to a newly identified gain-of-function mutation of the *Npr2* gene, p.Val883Met. Increased levels of cGMP both in blood samples of the patients and whole cell lysates expressing the mutant receptor clearly indicate its hypermorphism. Transgenic mice in which p.Val883Met mutant NPR2 was expressed in chondrocytes exhibited the elevated cGMP concentration and the excessive growth and deformities of vertebrae and long bones, which reproduced the symptoms observed in the patients. Taken together, our data are compatible with the interpretation that p.Val883Met mutant is constitutively active and causes skeletal overgrowth by increasing the level of cGMP in chondrocytes. However, we cannot exclude the possibility that the high expression level of NPR2 also contributed to overgrowth in transgenic mice to some extent.

Histological examination confirmed that the skeletal overgrowth was caused by the widening of the growth plates in the transgenic mice expressing the mutant *Npr2* (Fig. 5). As expected, the histological finding was similar to, but severer than, that reported in the mice with overexpression of CNP in chondrocytes [14]. The disorganized arrangement of chondrocytes in the transgenic mice indicates the critical role of the CNP/NPR2 pathway in chondrogenesis. Interestingly, the number of hypertrophic chondrocytes appeared to be increased in the transgenic mice, suggesting the involvement of CNP/NPR2 pathway in chondrocyte differentition.

Although NPR2 is expressed in various tissues, the phenotype seems to be confined to cartilage and bone in humans, as suggested by the clinical manifestations of our patients. It is worth noting that the transmission of the mutant *Npr2* gene seems to have had no apparent effect on fertility in the family, although infertility was observed in *Nppc* or *Npr2* null or hypomorphic mice [7], [15].

The second messenger cGMP is generated by two distinct types of guanylyl cyclases; cytoplasmic (soluble) and membrane-bound [16]. The two types share a similar catalytic domain which is conserved from bacteria to humans [17]–[18]. Seven mammalian membrane-bound guanylyl cyclases have been identified, consisting of ligand-binding, kinase homology, dimerization, and guanylyl cylase domains [4]. ANP, BNP, CNP, and intestinal peptides such as guanylin and uroguanylin have been identified as ligands for the membrane-bound guanylyl cylases. Although a few constitutively activate guanylyl cyclases were synthesized by chemical methods [19], p.Val883Met is the first gain-of-function mutation of NPR2 in living beings. Since cGMP is an important second messenger of several bioactive factors, cGMP-related drugs such as inhibitors of cyclic nucleotide phosphodiesterase have been developed. Erectile dysfunction, pulmonary dysfunction, cardiomyopathy, headache, dementia, and cancer are the targets of treatment with drugs that increase cGMP levels. However, drugs that directly activate guanylyl cylases have not been developed, with the exception of BAY 63-2521 (riociguat) [20]. The amino acid Val883 is located in the catalytic domain and preserved across species. The mechanism of activation in the mutant is beyond the scope of this paper, but the substitution may facilitate a conformational change in the catalytic domain, and in future, an analysis of the higher-order structure of the mutant protein may lead to the development of new drugs stimulating production of cGMP *in vivo*.

In conclusion, we have identified a gain-of-function mutation of *Npr2* in a family with tall stature and macrodactyly. Our findings reconfirm a critical role for NPR2 in skeletal growth in both humans and mice, and may provide a potential target for prevention and treatment of diseases caused by impaired production of cGMP.

## Materials and Methods

### Mutation Analysis

The mutation analysis was approved by the ethics committee at Osaka University Graduate School of Medicine, and written informed consent was obtained from the proband and family members for the analysis of the natriuretic peptide precursor C (*Nppc*), *Npr2*, *Npr3* and *Fgfr3* genes.

All the exons of the 4 genes were amplified using specific primers flanking the intron-exon boundaries according to published human *Nppc*, *Npr2*, *Npr3*, and *Fgfr3* genomic DNA sequences (UCSC genome browser: uc002vsl.1 at chromosome 2, 232498379–232499203; uc003zyd.1 at chromosome 9, 35782406–35799728; uc003jhv.2 at chromosome 5, 32711665–32787252; uc003gds.2 at chromosome 4, 1764337–1780396, respectively). Two primers for the *Nppc* gene, 14 primers for the *Npr2* gene, 9 primers for the *Npr3* gene, and 18 primers for the *Fgfr3* gene were designed using the software Primer3Plus and synthesized to cover the genomic sequence from the start codon to the termination codon. The sequences of the primers are provided in Tables 1, 2, 3, and 4. Polymerase chain reaction (PCR) products were sequenced using a Big Dye terminator cycle sequencing kit (version 3.1; Applied Biosystems) and an ABI 3130 automated sequencer (Applied Biosystems).

### Measurement of Serum Amino-Terminal proCNP Concentrations

Blood samples of III-1, II-5, I-2, and I-3 were centrifuged as soon as collected (15 min at 2,000×g, 4°C). The serum was stored at −80°C prior to use. NT-proCNP was assayed using an enzyme immunoassay (BIOMEDICA) according to the instructions provided. As a control, samples from 10 healthy adults (5 males and 5 females) were also measured.

### Construction of Expression Plasmids

The pcDNA3.1(+)/hemagglutinin (HA)-tagged human NPR2 wild-type vector (HA-WT) was a gift from Dr. Yoshihiro Ogawa (Tokyo Medical and Dental University, Japan) [21]. The construct encoding the mutant p.Val883Met, pcDNA3.1(+)/HA-human NPR2 Val883Met (HA-Val883Met), was generated by PCR-based mutagenesis using HA-WT as the template, and primers containing the nucleotide change. All the vector constructs were verified by bidirectional DNA sequencing.

### Cell Culture and Transfection

HEK293A cells were cultured in Dulbecco’s modified Eagle’s medium (DMEM) (Nacalai tesque) supplemented with 10% fetal bovine serum, 0.1 mM non-essential amino acid, and 2 mM L-glutamine at 37°C with 5% CO_2_. HEK293A cells were plated at a density of 1×10^5^ cells/12-well plate and cultured one day so as to reach confluence. Transfection was performed using the liposomal transfection reagent FuGENE6 (Reagent : DNA = 3 µl : 0.5 µg, Roche), according to the manufacturer’s instructions. The cells were used for the experiments 48 hours (h) after transfection.

### Immunoblot Analysis

The transfected cells were scraped into lysis buffer, sonicated for 2–3 sec and centrifuged to harvest the supernatant as the whole-cell extract. Five micrograms of protein was fractionated on a 10% sodium dodecyl sulfate (SDS)-polyacrylamide gel and blotted to a nitrocellulose membrane. A mouse monoclonal antibody against HA-tag (6E2, 1∶1000; Cell Signaling Technology) was used as the primary antibody. The signals were detected using SuperSignal West Dura Extended Duration Substrate (Thermo SCIENTIFIC) with a horseradish peroxidase-linked sheep anti-mouse IgG antibody (1∶10,000; Promega). As an internal control, β-actin in each sample was detected with a monoclonal anti-β-actin antibody (1∶4000; Sigma).

### Assay for cGMP of Transfected Cells and Plasma Samples

Transfected cells were serum-starved for 24 h before the cGMP assay and then incubated at 37°C with 5% CO_2_ in DMEM containing 0.5 mM IBMX (3-isobutyl-1-methylxanthine) (Wako) for 10 min. The cells were next treated with 1×10^−8^–1×10^−6^ M CNP-22 (Biochem) or vehicle (water) and incubated for another 10 min. The reaction was terminated with 300 µl of 0.1 M HCl, and the cGMP concentration was measured by a competitive enzyme immunoassay (Cayman Chemical). Results are presented as the mean ± SD. Student’s *t* test was used for statistical analyses.

Plasma cGMP concentrations of III-1, II-5, I-2, I-3, and control samples were measured with a cyclic GMP radio immunoassay kit (YAMASA).

### Generation of the Transgenic Mice Expressing the p.Val883Met Mutant NPR2 in Chondrocytes

For the generation of transgenic mice expressing the mutant NPR2 (p.Val883Met) specifically in cartilage, we used the p742-Int vector (constructed by Tsumaki. et al.) [22], containing the promoter and intronic enhancer of the *Col11a2* gene. The cDNA encoding the mutant *Npr2* was obtained from pcDNA3.1(+)/HA -Val883Met, and inserted into the *Not I* site of p742-Int. The transgene was gel-purified and microinjected into the pronuclei of 250 fertilized eggs from C57BL/6 mice. For genotyping, genomic DNA from the tail was subjected to PCR.

The ethical treatment of animal protocol was approved by the Committee on the Ethics of Animal Experiments of the Osaka University Graduate School of Medicine (Permit Number J004081-003). All surgery was performed under isoflurane anesthesia, and all efforts were made to minimize suffering.

### Identification of Transgenic Founders

RNA samples were extracted from tail samples containing cartilage tissue. The end of the tail (10 mm) was cut off, immediately placed in RLT lysis buffer (QIAGEN), fractured by MicroSmash MS-100 (TOMY; 4800 rpm, 19 sec with φ5 mm zirconia-ball), and centrifuged at 15,000 rpm for 3 min. Total RNA was extracted from the supernatant using an RNeasy kit (QIAGEN). RNA was reverse-transcribed using the SuperScript III CellsDirect cDNA Synthesis System (Invitrogen). Real-time PCR was performed using SYBR® Green I fluorescence (Roche). Data were analyzed with LightCycler analysis software, version 3.5 (Roche). The amount of transgene mRNA was standardized based on that of murine *Sox9* mRNA, which is specifically expressed in chondrocytes. The transgene and murine *Sox9* primer sets for real-time PCR are listed in Table 5.

### Determination of the Npr2 mRNA Amount in Mouse Cartilage

RNA samples were isolated from the costal cartilage of line No.28 offspring (Tg. 28) and wild-type littermates (Wt) at postnatal day 5 under a stereomicroscope. For RNA extraction, the samples were treated as previously explained and real-time PCR was performed. The total amount of *Npr2* mRNA, which contains the sequence of mRNA from exon 15 to 16, and has complete commonality between humans and mice, was standardized based on that of murine *Gapdh* mRNA. The total *Npr2* and murine *Gapdh* primer sets for real-time PCR are listed in Table 5. Student’s *t* test was used for statistical analyses.

### Assay for cGMP in Mouse Cartilage

The costal cartilage of Tg. 28 and Wt at postnatal day 5 was isolated under a stereomicroscope. The samples were immediately placed in 5% trichloroacetic acid (Wako), fractured by MicroSmash MS-100 and centrifuged at 15,000 rpm for 3 min. The cGMP concentration was measured by a competitive enzyme immunoassay (Cayman Chemical) according to the manufacturer’s directions. The pH of the cGMP standard and samples was neutralized with a phosphate buffer as previously described [23]. cGMP concentrations were standardized based on the DNA concentration of each sample, measured by an ultraviolet vision spectrophotometer for nucleic acid (NanoDrop 2000, Thermo SCIENTIFIC). Student’s *t* test was used for statistical analyses.

### Soft X-ray Analysis of the Transgenic Mice

A soft X-ray analysis of the skeleton was performed using SOFTEX M-60 (30 kV, 3 mA, 80 sec, Softex), and faxitron X-ray (35 kV, 120 sec, Faxitron x-ray Corp).

### Histological Analysis

Femurs were harvested, fixed in 4% paraformaldehyde, and decalcified in EDTA solution. Sections were cut from paraffin-embedded specimens of knee-joint, and stained with hematoxylin-eosin (H&E).
